# A mini-review of the evidence for cerebrovascular changes following gender-affirming hormone replacement therapy and a call for increased focus on cerebrovascular transgender health

**DOI:** 10.3389/fnhum.2023.1303871

**Published:** 2023-11-22

**Authors:** Melissa Emily Wright, Kevin Murphy

**Affiliations:** Cardiff University Brain Research Imaging Centre (CUBRIC), School of Psychology, College of Biomedical and Life Sciences, Cardiff University, Cardiff, United Kingdom

**Keywords:** hormone, cerebrovascular, transgender, hormone replacement therapy (HRT), LGBT, vascular health

## Abstract

Gender-affirming hormone replacement therapy (gaHRT) is an important step for many in the gender diverse community, associated with increased quality-of-life and lower self-reported scores of depression and anxiety. However, considering the interactions that the involved sex hormones have on vasculature (with oestrogen and testosterone demonstrating vasodilatory and vasoconstricting properties, respectively), it is important for transgender healthcare research to examine how the manipulation of these hormones interact with cerebrovascular structure and functioning. There is a stark lack of research in this area. This mini-review outlines the research suggesting a vascular impact of these sex hormones using evidence from a range of cohorts (e.g., menopause, polycystic ovary syndrome) and discusses the work that has been done into cerebrovascular changes following gaHRT. Finally, recommendations for future research into cerebrovascular health in transgender cohorts following gaHRT are outlined.

## Introduction

Transgender people are individuals whose gender identity does not align with that assigned to them at birth. While exact prevalence rates of gender diverse individuals are difficult to determine due to the variety of criteria and methods used (Collin et al., 2016), the self-reported rate is ∼0.1–2%, depending on geographical location (Goodman et al., 2019; Spizzirri et al., 2021). Despite this, it represents an under-researched population. For example, only 0.08% of clinical trials published between January 2018 and July 2022 reported transgender participation (Round et al., 2023).

Transgender people may take gender-affirming hormone replacement therapy (gaHRT) to aid in their gender transition. This forms an essential part of people’s transition (Mohamed and Hunter, 2018), improving anxiety and depression and alleviating symptoms of gender dysphoria (see Table 1 for definitions; Colizzi et al., 2013; Baker et al., 2021; Chaovanalikit et al., 2022). To better understand and serve transgender health, it is important to consider the impact of gaHRT treatment.

**TABLE 1:** List of definitions.

Definitions	
*gaHRT*	Gender-affirming hormone replacement therapy—a therapy taken as part of a gender transition that aids in feminising or masculinising bodies to align with their gender identity. It should be noted that this can be taken by transgender men, transgender women, or non-binary individuals who may or may not identify with the descriptor “transgender.”
*Transgender person*	A person whose gender identity does not align with that assigned to them at birth.
*Cisgender person*	A person whose gender identity does align with that assigned to them at birth.
*Gender dysphoria*	A feeling of intense distress that can occur when an individual’s biological sex at birth does not align with their gender identity. This doesn’t occur in all transgender individuals but can be associated with anxiety and depression.
*Gender euphoria*	In contrast to the above, this is the feeling of joy or “rightness” when one’s presentation aligns with their gender identity.
*CBF*	Cerebral blood flow
*CBV*	Cerebral blood volume
*CVR*	Cerebrovascular reactivity

This review focuses on the influence of gaHRT in transgender individuals on cerebrovascular function and health. Measures of cerebrovascular function include cerebral blood flow (CBF), blood-brain barrier function, cerebral blood volume (CBV), cerebrovascular reactivity (CVR), and cerebral vessel pulsatility. The brain has a high metabolic demand, and the supporting vasculature is essential for maintaining cognitive function and brain health (Iadecola, 2017; Zimmerman et al., 2021). As hormones can have large vascular effects, it is vitally important to investigate how gaHRT influences cerebrovascular health to inform transgender healthcare.

## Gender-affirming hormone replacement therapy

There are two broad categories of gaHRT. Masculinising gaHRT introduces exogenous testosterone to maximise virilization (e.g., deepening of the voice, changes in musculature, hair growth) and to suppress female secondary sex characteristics. A range of administration routes are available (oral, subcutaneous, transdermal) to achieve blood concentrations of 300–1,000 ng/dL (after which, the goal becomes maintenance; Unger, 2016). While the early presentation of masculinising effects is dose-dependent, different dosages appear equally effective after six-months (Nakamura et al., 2013).

Feminising gaHRT involves the delivery of exogenous oestrogen which changes fat distribution and reduces male pattern hair growth (Giltay and Gooren, 2000). This suppresses androgen production (Dittrich et al., 2005) and is generally prescribed with additional anti-androgenic therapy. Different administration routes are available (Oral, subcutaneous, transdermal) to achieve the blood oestradiol target level of 100–200 pg/mL (Unger, 2016). The addition of progesterone to gaHRT treatment regimens, to better represent female hormonal profiles, may be beneficial for feminising outcomes, but it’s efficiency remains under-researched (Deutsch, 2016; Prior, 2019; Milionis et al., 2022). In both masculinising and feminising cases, the exact form and dosage of gaHRT can and should be tailored to an individual and their transition goals.

Gender-affirming hormone replacement therapy (gaHRT) benefits wellbeing and health outcomes (e.g., depression, anxiety, quality-of-life, self-reported and physiological stress) equally across gender identity, age and psychological attachment style (e.g., Colizzi et al., 2013; Baker et al., 2021; Chaovanalikit et al., 2022).

It is important to consider how an altered hormonal profile will influence cerebral vasculature structure and function. Generalising from other populations is inadequate; transgender individuals may present with different healthy reference levels than their cisgender counterparts (e.g., Roberts et al., 2014, found that “normal” levels of certain clinical measurands, important for diagnosis and monitoring, was different following gaHRT compared to cisgender individuals of either sex) and interaction with other factors (e.g., age of onset, length of use, previous hormonal history, environmental stress) may lead to altered patterns of risk.

## The influence of sex hormones on cerebrovascular health—evidence from other cohorts

The importance of investigating cerebrovascular health after hormonal changes can be highlighted by examining other cohorts. Although these results may not generalise, they demonstrate that sex hormones influence cerebrovascular health, suggesting that effects may be expected following gaHRT.

### Oestrogen and progesterone

Oestrogen receptors are found throughout the brain (Shughrue and Merchenthaler, 2001; Milner et al., 2010; Mitterling et al., 2010) and the hormone is considered to be vascular- and neuro-protective. Cisgender women have a lower risk of cardiovascular and cerebrovascular disease compared to cisgender men up until menopause, at which point their risk significantly increases (Hayward et al., 2000; Aggarwal et al., 2018). Earlier menarche and longer reproductive lifespan are also associated with lower risk of stroke, suggesting a beneficial influence of lifetime exposure to oestrogen on the cerebrovascular system [Chen et al., 2023; relatedly, lifetime oestrogen exposure also shows associations with better memory score and larger medial temporal cortical volumes (Steventon et al., 2023)]. Animal models demonstrate that oestrogen increases vasodilation (Tostes et al., 2003), decreases pathological vasoreactivity (Wassmann et al., 2001), and increases CBF and angiogenesis (Robison et al., 2019). Additionally, it suppresses inflammatory responses and increases perfusion after ischemic injury (Hurn et al., 1995; Santizo et al., 2002). This vascular-protective effect is present in both male and female animal models (Roof and Hall, 2000), though may be age-dependant (Deer and Stallone, 2016).

The influence of progesterone on the cerebrovascular system is less clear, with conflicting results on inflammation. For example, Gibson et al. (2005) reports that administration of progesterone suppressed aspects of the post-injury inflammatory response, while Sunday et al. (2006) found that the administration of progesterone exacerbated the inflammatory response in ovariectomized rats. However, there have been multiple reports of progesterone having a beneficial impact on recovery from brain injury (Chen et al., 2011, 2021; Gibson et al., 2011) and facilitating vascular reactivity (Cunha et al., 2020; da Costa et al., 2021).

Due to relatively predictable lifetime changes in oestrogen and progesterone, there are several informative cohorts for investigating their combined influence on the human cerebrovascular system. By assessing cisgender women multiple times across a menstrual cycle, oestrogen and progesterone were found to have independent, region-specific influences on CBF (Cote et al., 2021). Fluctuations in progesterone and oestrogen/progesterone ratio impact CVR (Debert et al., 2012; Skinner et al., 2023). While some studies report reduced CBF and CVR following menopause, high-quality evidence and consistent menopausal criteria are lacking (Ruediger et al., 2021). A confounding factor is the impact of age and physical fitness at menopause age on cerebrovascular health (Chen et al., 2023; Moir et al., 2023; Ruediger et al., 2023). A recent systematic review of the influence of sex hormones on cerebrovascular function in human subjects suggested that, though there were often differences between high and low hormonal states, the directions were not consistent (Skinner et al., 2021). A meta-analysis indicated that HRT improves pulsatility but not CBF, though notable heterogeneity exists (Skinner et al., 2021). More research is needed to illustrate the influence of oestrogen and progesterone on cerebrovascular function. While a vascular impact is strongly suggested, mainly by animal research, the interactions are complex and may be influenced by factors unique to each cohort (e.g., current age, age of menarche, chronic vs. acute exposure/deprivation and history of HRT use).

### Testosterone

Testosterone is a prominent circulating androgen affecting receptors throughout the neural system (Sheridan, 1983; Sarkey et al., 2008), with its potency linked to genetic factors (Chamberlain et al., 1994; Tirabassi et al., 2013). Animal model and isolated cell culture studies suggest that androgen levels improve angiogenesis (Louissaint et al., 2002; Yoshida et al., 2013), though this effect is diminished with age (Lecce et al., 2014). Testosterone appears to influence pathways that mediate CBF, with chronic testosterone deprivation/exposure in male animal models eliciting vasodilation/vasoconstriction of cerebral vessels, respectively (Geary et al., 2000; Gonzales et al., 2004, 2005; Abi-Ghanem et al., 2020). This vasoconstrictive influence may explain cisgender sex differences in CBF, which is higher in women than men (e.g., Rodriguez et al., 1988; Aanerud et al., 2017). However, acute testosterone administration in other vascular beds elicits vasodilation (Yue et al., 1995; Deenadayalu et al., 2001; Tep-areenan et al., 2002). At a cellular level, testosterone facilitates vasodilatory and protective mechanisms (Deenadayalu et al., 2001; Perusquía, 2003; Perusquía et al., 2015). Testosterone’s influence may also be mediated by sex, possibly due to hormonal history or other sex-associated differences in vascular physiology. Sieveking et al. (2010) found that androgen administration increased angiogenesis only in male mice models. Additionally, testosterone levels appear to have opposite actions on ET-1 (a vasoconstrictor) in cisgender vs. transgender men (Polderman et al., 1993; Kumanov et al., 2007; Abi-Ghanem et al., 2020). While testosterone appears to have a vasoconstricting influence on the vascular system, the effect is mediated by multiple other factors (such as sex and age), and it may facilitate vasodilation in certain vessels.

Testosterone’s influence on cerebrovasculature in cisgender women or following gaHRT is less well known. An important cohort for investigating this is patients with polycystic ovary syndrome (PCOS), a condition commonly characterised by hyperandrogenism, irregular/missing menstruation, and ovarian cysts. Excess testosterone increases systemic arterial stiffness in PCOS patients compared to controls (Kilic et al., 2021) but the influence on cerebrovascular function is vastly under-researched. Acar et al. (2005) reported no statistically significant difference in CBV using colour duplex sonography, though blood velocity in the vertebral artery was significantly reduced in PCOS patients. Direct comparisons to testosterone level were not made. The PCOS cohort represents an excellent opportunity to investigate testosterone’s influence on the cerebrovascular system.

It is noteworthy that testosterone can be locally metabolised into oestrogens, making each hormone’s influence difficult to separate. For example, estrone level, a form of oestrogen, is elevated in PCOS patients (DeVane et al., 1975).

## Vascular research in transgender populations undergoing gaHRT

Research specifically investigating cerebrovascular health in transgender populations undergoing gaHRT has focused on disease outcomes rather than function (e.g., CBF, CVR). There exists an increased risk of ischemic stroke following feminising gaHRT (Connelly et al., 2019; Pribish and Iwamoto, 2023), though the low event rate present in younger cohorts limits interpretation (e.g., of 966 transgender women, five died from stroke; Asscheman et al., 2011). The use of an outdated oral form of gaHRT (ethinyl oestradiol) in these studies also limits the present-day generalisability of the results (Asscheman et al., 2011; Irwig, 2018; Pribish and Iwamoto, 2023). Current, but not past, ethinyl oestradiol use is linked to cardiovascular events (Asscheman et al., 2011). However, ischemic stroke risk is increased in transgender women, even when excluding ethinyl oestradiol use (Getahun et al., 2018). Notably, the cerebrovascular event risk was unchanged initially and only increased after the 6 year follow-up (Getahun et al., 2018), which highlights the importance of longitudinal comparisons. While Getahun et al. (2018) found elevated cerebrovascular disease risk in transgender women compared to cisgender women (but not cisgender men), others report the opposite pattern (Wierckx et al., 2013) or no difference at all (Meyer et al., 2017), suggesting unappreciated mediating factors. Cerebrovascular disease risk is not elevated with masculinising gaHRT (Wierckx et al., 2013; Getahun et al., 2018; Connelly et al., 2019) and in fact migraine risk (related to vasculature) appears lower (Todd et al., 2023). In terms of broader cardiovascular disease outcomes and risk factors, a similar pattern is seen, with elevated risk in transgender women but not transgender men (Connelly et al., 2019; Kulprachakarn et al., 2020; Pribish and Iwamoto, 2023). However, this is not always observed (Martinez et al., 2023) and an improvement in vascular function following feminising gaHRT has been reported (New et al., 1997, 2000). Importantly, Karalexi et al. (2022) reported that cardiovascular disease incidence in gender diverse populations was similar regardless of gaHRT use. Increased disease incidence may therefore be due to healthcare barriers (Safer et al., 2016) and other environmental factors (e.g., minority stress) rather than gaHRT. Clearly, more work is needed to understand risk following gaHRT, especially following feminising gaHRT, which mirrors the mixed evidence of vascular benefits following post-menopausal HRT despite protective effects of oestrogen (Boardman et al., 2015). More research into specific cerebrovascular functions may help in understanding this complex relationship.

Evidence for changes in cerebral vessels can also be inferred from related work. A recent review describes twenty transgender male patients who had been undergoing gaHRT and presented with intracranial hypertension, a condition characterised by increased intracranial pressure (ICP). They suggest an association with cerebral spinal fluid (CSF) hypoandrogenism (Kamboj et al., 2023). Chronic testosterone treatment in lean rat models led to an increase in ICP, potentially due to increased CSF secretion rate (Wardman et al., 2023). ICP itself is associated with vascular pulsatility, an index of vascular health (Hamzah et al., 2020). Increased vascular pulsatility is associated with damage to cerebrovascular microstructure and cognition due to pulsatile stress reaching the brain (Singer et al., 2014; Palta et al., 2019). If cerebrovascular pulsatility is altered by testosterone-based gaHRT, this may be an important area for monitoring/consideration. Cunha et al. (2023) found that arterial stiffness (measured using carotid–femoral pulse wave velocity) was significantly higher in transgender men compared to cisgender men and women, suggesting increased aortic stiffness and thus increased levels of vascular pulsatility. Within the transgender men group, there was a significant positive correlation between gaHRT duration (which ranged from 4 to 32 years) and pulsatility. Such studies provide important insights into how masculinising gaHRT impact vascular haemodynamics. Future studies will shed light on how this translates to cerebral vasculature and brain health.

A small number of studies have investigated the retina, the layer of neural tissue in the anterior eye that supports vision. The retina is of interest because it can be more directly imaged non-invasively than the cerebrovasculature. Changes in retinal vasculature and structure are associated with many vascular disorders (e.g., Sairenchi et al., 2011; Hanff et al., 2014; Moss, 2015; Wiseman et al., 2023). Measuring blood flow in the ophthalmic artery using Colour Doppler Ultrasonograph, Alpogan et al. (2021) observed no difference between transgender men, cisgender men, and cisgender women. However, the systole/diastole flow ratio significantly correlated with circulating testosterone level. Using Optical Coherence Tomography Angiography (OCT-A), Tüten et al. (2022) found that retinal vessel density (VD) was lower across multiple regions in transgender men compared to cisgender women controls, reaching statistical significance in the inferior region and for radial peripapillary capillary VD. These studies provide important information on the influence of exogenous testosterone on ocular health and may suggest wider vascular changes. However, without transgender women participants as a comparison, conclusions about whether it is the testosterone *per se* or just hormonal profile changes are difficult to draw. For example, while oestrogen is reported to be protective of vascular health (e.g., Parker et al., 2009; Burns and Korach, 2012; Iorga et al., 2017; Novella et al., 2019; Shin et al., 2022), oestrogen (or combined oestrogen and progesterone) HRT does not conclusively prevent cardiovascular disease in post-menopausal women (Boardman et al., 2015). Overall, the retina provides a unique window into general cerebrovascular health and specifically neural microvasculature.

In conclusion, cerebrovascular function changes in transgender populations taking gaHRT. However, the research into disease outcomes and risk paints a complex picture that is not fully understood. Cerebrovascular function needs to be fully investigated against detailed hormonal profiles.

## Recommendations

More research is needed in this area. Cohort-relevant factors such as age of onset, previous hormonal profile, length of use, and environmental stresses must be considered. In particular, longitudinal studies with long-term follow ups would be beneficial as gaHRT may be taken for multiple decades (Mohamed and Hunter, 2018). Though such studies represent a significant investment, they are essential in determining how cerebrovascular health is affected by long-term hormonal use. Additionally, cross-sectional studies should take into account many confounding factors that will vary between transgender and cisgender participants, such as chronic stress, lifestyle, level of dysphoria, external support systems, and traumatic experiences. Transgender cohorts represent a marginalised group and face significant social stressors in their day-to-day life (Harrison et al., 2012; Valentine and Shipherd, 2018; Chodzen et al., 2019; Lin et al., 2021; Wilson et al., 2023), which themselves are associated with cerebrovascular function changes (Endo et al., 1999; Lee et al., 2015; Burrage et al., 2018). Consideration of such factors is also important during longitudinal studies, as reported levels of psychological wellbeing improve over the course of gaHRT (e.g., Chaovanalikit et al., 2022). Well-controlled and considered longitudinal studies are essential in determining the influence of gaHRT on cerebrovascular health.

Older adults undergoing gaHRT are a particularly under-researched subgroup. The cerebrovascular benefits/risks in older people who commenced gaHRT at a young age may present very differently to those who start gaHRT in later life, especially considering age-related comorbidities. Qualitative research into transgender women aged 20–79 years old (96% of which were taking gaHRT) suggest that, although it is an “essential” part of their transition, they have concerns about the long-term effects of gaHRT (Mohamed and Hunter, 2018). Research should consider how to best serve the community and address such concerns (Minalga et al., 2022).

It should also be noted that many studies involving transgender individuals use relatively small sample sizes and convenience sampling. They therefore may mostly consist of a narrow range of demographics (e.g., university educated, secure socioeconomic status). To recruit a broader and more representative sample, the transgender community must be actively engaged with. Some of the keys barriers for research participation reported by transgender persons were “lack of trust in research” and “lack of knowledge of opportunities” (Owen-Smith et al., 2016). There are also more cohort-specific worries that should be considered for the safety and comfort of participants, such as the risk of being “outed” (i.e., having their identity shared or obvious to external individuals; Owen-Smith et al., 2016). This particular point could be addressed by, for example, highlighting any relevant signage, being clear about the study location, and outlining anonymisation practises in participant information sheets. Active engagement and public awareness campaigns will be essential for increasing diverse participation and rebuilding trust, as well as gaining input from transgender individuals at the study design stage. Larger and more representative samples will allow for more confident and relevant conclusions to be drawn.

The language of research into the general population can be easily adjusted to be more inclusive to the gender-diverse community and therefore produce more representative results. For example, allowing participants to define their own gender rather than select from a binary response, provides a much richer and more representative dataset (Ghorbanian et al., 2022). Additionally, adjusting the language of calls for participation, surveys and study documents may help in making research more approachable to a wider variety of participants, reducing bias and improving the accessibility/inclusivity of participation pools.

Finally, adequately controlled studies are important. For example, some studies found significant differences when comparing transgender women to cisgender women but not cisgender men (Getahun et al., 2018), while others report the opposite pattern (Wierckx et al., 2013). Studies that only include one cisgender control group may be missing a larger pattern. Additionally, by including both feminising and masculinising gaHRT groups, researchers can make better inferences about whether fluctuations in an outcome metric is due to a particular hormone *per se*, or just the act of altering hormonal profiles in general.

## Conclusion

Significant progress is needed to determine the impact of gaHRT on transgender participants’ cerebrovascular health to uncover mechanisms behind cerebrovascular or neurodegenerative disease risk (Brady et al., 2023). Such studies may highlight subtle risk patterns and will uncover how sex hormones interact with the cerebrovascular system in general populations. It is important to investigate cerebral vessels specifically, as the vasodilatory properties of oestrogen can vary by vascular bed location (Opgaard et al., 2002). In the future, long-term longitudinal research with considered controls and inclusive language will allow for more comprehensive, informative, and representative conclusions to be drawn. As mentioned by Pribish and Iwamoto (2023), work suggesting a vascular risk should not dissuade people from gaHRT use, but instead direct patient education and management to vascular health, highlighting modifiable risk factors before and during gaHRT use.

## Author contributions

MW: Conceptualization, Writing – original draft. KM: Conceptualization, Writing – review and editing.

## Acknowledgements

We would like to thank Wellcome Trust for their help in publication of this article.
